# Severe Fever with Thrombocytopenia Syndrome in Cats and Its Prevalence among Veterinarian Staff Members in Nagasaki, Japan

**DOI:** 10.3390/v13061142

**Published:** 2021-06-14

**Authors:** Tsuyoshi Ando, Takeshi Nabeshima, Shingo Inoue, Mya Myat Ngwe Tun, Miho Obata, Weiyin Hu, Hiroshi Shimoda, Shintaro Kurihara, Koichi Izumikawa, Kouichi Morita, Daisuke Hayasaka

**Affiliations:** 1 Department of Virology, Institute of Tropical Medicine, Nagasaki University, Nagasaki 852-8523, Japan; tsuyoshi.ando@hugp.com (T.A.); mtmikami@tm.nagasaki-u.ac.jp (T.N.); pampanga@nagasaki-u.ac.jp (S.I.); myamyat@tm.nagasaki-u.ac.jp (M.M.N.T.); moritak@nagasaki-u.ac.jp (K.M.); 2 Program for Nurturing Global Leaders in Tropical and Emerging Communicable Diseases, Graduate School of Biomedical Sciences, Nagasaki University, Nagasaki 852-8523, Japan; 3 Laboratory of Veterinary Microbiology, Joint Faculty of Veterinary Medicine, Yamaguchi University, 1677-1 Yoshida, Yamaguchi 753-8511, Japan; g009tb@yamaguchi-u.ac.jp (M.O.); w010tb@yamaguchi-u.ac.jp (W.H.); hshimoda@yamaguchi-u.ac.jp (H.S.); 4 Department of Medical Safety, Nagasaki University Hospital, Nagasaki University, Nagasaki 852-8501, Japan; kurihiro@nagasaki-u.ac.jp; 5 Infection Control and Education Center, Nagasaki University Hospital, Nagasaki University, Nagasaki 852-8501, Japan; koizumik@nagasaki-u.ac.jp; 6 Department of Infectious Diseases, Graduate School of Biomedical Sciences, Nagasaki University, Nagasaki 852-8523, Japan

**Keywords:** severe fever with thrombocytopenia syndrome, cats, veterinarians

## Abstract

In this study, we investigated severe fever with thrombocytopenia syndrome (SFTS) virus (SFTSV) infection in cats in Nagasaki, Japan. In total, 44 of 133 (33.1%) cats with suspected SFTS were confirmed to be infected with SFTSV. Phylogenetic analyses of SFTSV isolates from cats indicated that the main genotype in Nagasaki was J1 and that unique reassortant strains with J2 (S segment) and unclassified genotypes (M and L segments) were also present. There were no significant differences in virus growth in cell cultures or fatality in SFTSV-infected mice between the SFTSV strains that were isolated from recovered and fatal cat cases. Remarkably, SFTSV RNAs were detected in the swabs from cats, indicating that the body fluids contain SFTSV. To evaluate the risk of SFTSV infection when providing animal care, we further examined the seroprevalence of SFTSV infection in veterinarian staff members; 3 of 71 (4.2%) were seropositive for SFTSV-specific antibodies. Our results provide useful information on the possibility of using cats as sentinel animals and raised concerns of the zoonotic risk of catching SFTSV from animals.

## 1. Introduction

Severe fever with thrombocytopenia syndrome (SFTS) was first reported in the central part of China [1] and has subsequently been reported in Korea, Japan, Taiwan, and Vietnam [2,3,4,5,6]. In addition, detections of anti-SFTS virus (SFTSV) antibodies and SFTSV RNA in humans have indicated the presence of SFTSV in Myanmar and Pakistan [7,8].

The causative agent of SFTS is the SFTSV, which belongs to the order Bunyavirales, family Phenuiviridae, genus *Bandavirus*, and species *Dabie bandavirus*. The viral genome consists of three segments, the large (L) (negative-sense genome), medium (M) (negative-sense genome), and small (S) (ambisense genome) segments, which encode RNA-dependent RNA polymerase (RdRp), glycoproteins (Gn and Gc), and a nucleoprotein (N) and non-structural protein (NSs), respectively [1].

SFTSV is transmitted to humans and animals through the bite of infected ticks, such as *Haemaphysalis longicornis* [9]. Seroepidemiological surveys have shown the presence of anti-SFTSV antibody-positive animals, including wild boars, sheep, cattle, cats, and dogs, in SFTS-endemic areas [10,11,12,13,14,15], suggesting that SFTSV circulates between ticks and animals in nature. Therefore, epidemiological studies in ticks and sentinel animals are expected to provide important information on the distribution of SFTSV in the endemic areas.

In human cases, the clinical symptoms of SFTS include fever, enteritis, thrombocytopenia, and leukopenia, and the fatality rate has been reported to be as high as 30% [2,16,17,18]. No specific treatments or vaccines for SFTS are currently available, although the prevention of disease spread is currently the main priority.

As of December 2020, 573 SFTS cases have been identified in Japan, in the western part of the country. In addition, there have been recent reports of fatal cases of SFTSV in companion animals, including cheetahs, cats, and dogs [14,19,20,21]. Remarkably, veterinarians and pet owners may also have been infected by SFTSV via direct transmission from SFTSV-infected animals [22,23,24]. These findings raised concerns of zoonotic risk of SFTSV transmission from these animals.

Nagasaki, which is located on the Japanese island of Kyushu, is an endemic area of SFTS, and 40 cases of SFTS have been identified as of 2020; a retrospective study has reported that the earliest case of SFTS was discovered in Nagasaki in 2005 [25]. We have reported on epidemiological studies of SFTSV infections in ticks [26], seroepidemiological research in wild boars [13], and a prevalence survey in dogs (unpublished results) in Nagasaki.

In this study, we surveyed SFTSV infections in cats in Nagasaki to better understand the situation in companion animals. We also investigated the epidemiological distribution based on SFTSV isolates from cats and attempted to evaluate the pathogenic properties of the SFTSV isolates from them. In addition, to examine the possible risk of SFTSV infection when providing animal care, we examined the seroprevalence in veterinarians and nurses in Nagasaki. Our results provide useful information on the possibility of using cats as sentinel animals, the SFTSV distribution and molecular epidemiology in Nagasaki, and the possible zoonotic risk of SFTSV.

## 2. Materials and Methods

### 2.1. Cat Samples

**Figure 1:**
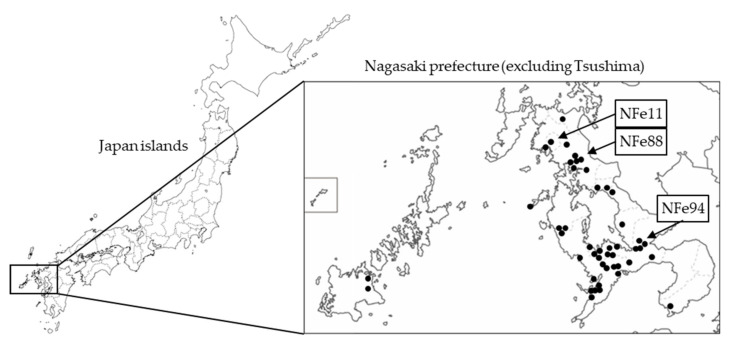
Location of Nagasaki in Japan and the distribution of the identified SFTS-positive cat cases. Closed circles indicate the places where SFTSV-positive cats were found. Arrows indicate the places where the NFe11 and NFe88 reassortant strains and the NFe94 strain (J2 genotype) were isolated.

With the support of the Nagasaki Veterinary Medical Association, samples from cats with suspected SFTS were provided by animal hospitals located in Nagasaki prefecture (Figure 1) between March 2018 and March 2020. Samples included sera, plasma, oral swabs, rectal swabs, and conjunctival swabs from 133 animals.

### 2.2. SFTSV RNA Detection

The *SFTSV* gene in cat samples was detected using real-time RT-PCR, described previously [26]. RNA was extracted using Isogen-LS (Nippon Gene, Tokyo, Japan), and the RT-PCR reaction was performed using a One-Step PrimeScript RT-PCR Kit (Takara Bio Inc. Shiga, Japan) and 7500 Real-time RT-PCR System (Applied Biosystems, Massachusetts, MA, USA). SFTSV-specific primers and a probe were designed based on a consensus sequence of the RdRp gene [26]. The copy numbers were calculated as the ratio of the copy numbers to the standard control prepared from a cloned plasmid vector [26].

### 2.3. SFTSV and Cells

Serum samples of SFTS-positive cats were intraperitoneally inoculated into A129 mice, and the spleens were collected when the mice exhibited clinical signs. The spleens were also used for further virus isolations and whole-genome sequencing of the SFTSV isolates. To obtain the infectious SFTSV, the samples were homogenized in phosphate-buffered saline and inoculated onto Vero E6 cells. After 6 to 7 days of incubation, the cell culture supernatants were obtained as stock viruses. The SFTSV YG-1 strain was kindly provided by Ken Maeda of the Institute of Infectious Diseases and Yamaguchi University. Vero E6 cells were maintained in Eagle’s minimal essential medium (EMEM) (Nissui Pharmaceutical Co. Tokyo, Japan) containing 10% fetal bovine serum (FBS).

The SFTSV titer was determined by a focus forming assay. Confluent Vero E6 cells were inoculated with serially diluted culture supernatants of the SFTSV stocks and incubated in EMEM with 2% FBS containing 1% methyl cellulose 4000 (Wako Pure Chemical Industries, Ltd., Osaka, Japan) for 5 days. Viral foci were detected using SFTSV antiserum, peroxidase-conjugated anti-human IgG (American Qualex, San Clemente, CA, USA), and DAB substrate (Wako Pure Chemical Industries, Ltd., Osaka, Japan). Viral titers were calculated as focus-forming unit (ffu)/mL.

All experiments using infectious SFTSV were performed in a biosafety level 3 laboratory at Nagasaki University according to standard BSL3 guidelines.

### 2.4. SFTSV Genome Sequence

RNA was extracted from the homogenized mouse spleens using Isogen-LS (Nippon Gene, Tokyo, Japan). The whole genomes of the SFTSV isolates from cats were determined using next-generation sequencing as described previously [27]. Phylogenetic trees were constructed for the S, M, and L segment genes using the maximum likelihood (ML) method with the GTRGAMMAI model using RAxML version 8.2. Viral sequences of novel isolates in this study were deposited in the GenBank database (accession nos MN995224 to MN995310). Guertu virus, which belongs to the family Phenuiviridae and genus *Bandavirus*, was used as an outgroup to construct each phylogenetic tree of S, M, and L segments.

### 2.5. Growth Curves of SFTSV in Vero E6 Cells

A total of 10^5^ cells per well of the Vero E6 cells were infected with 10^3^ ffu of each of the SFTSV isolates from cats at a multiplicity of infection of 0.01 in 12-well plates. The experiment was performed in triplicate. The culture supernatants were harvested daily up to 3 days post-infection, and the virus titers were determined.

### 2.6. SFTSV Infection in Mice

A129 mice were purchased from B & K Universal Ltd. (North Humberside, UK). The mice were mated in the animal facility at Nagasaki University. Adult mice older than 8 weeks of age were subcutaneously inoculated with 10^2^ or 10^6^ ffu of SFTSV diluted in EMEM containing 2% FBS. Mice were weighed daily and observed for clinical signs. The experimental protocols were approved by the Animal Care and Use Committee of Nagasaki University (approval numbers: 190212509-2, 5 September 2019; and 1902011507-2, 10 September 2019).

### 2.7. Serum Sample of Veterinarian Staff Members

With the support of Nagasaki Veterinary Medical Association, serum samples were collected from veterinarians and nurses in animal hospitals in Nagasaki. The study using human samples was approved by the ethics committee of the Nagasaki University Hospital, Nagasaki University (approval number: 13032781-3, 20 June 2019).

### 2.8. Enzyme-Linked Immunosorbent Assay (ELISA) for SFTSV-Specific IgG

Indirect ELISA for SFTSV-specific IgG was performed according to previously described methods with modifications [13]. Antigens prepared from the lysate of SFTSV-infected Huh-7 cells were kindly provided by Shigeru Morikawa of the National Institute of Infectious Diseases, Tokyo, Japan. The antigens were coated onto 96-well plates and were incubated at 4 °C overnight. After blocking, 1:100 diluted serum or plasma samples were applied and incubated at 37 °C for 1 h; then, the secondary antibody (anti-human IgG conjugated with horse radish peroxidase; 1:1000) was added. o-Phenylenediamine dihydrochloride (Sigma Chemical, St. Louis, MO, USA) dissolved in 50 mM citrate PBS containing 0.1% hydrogen peroxide was then added. Subsequently, 1 N sulfuric acid was added to terminate the reaction, and the optical density values were determined at 492 nm (Multiscan JX, model no. 353; Thermolab System, Tokyo, Japan). The cut-off OD value was determined as 2 times of the negative sample averages.

### 2.9. Neutralizing Test

The neutralizing antibody titers of the antiserum were determined by a focus-reduction assay [13]. Serially diluted sera were mixed with the SFTSV YG-1 strain and incubated at 37 °C for 1 h. Vero E6 cells were inoculated with the mixtures and incubated for 5 days. Focus staining was performed as described above. The neutralizing titer was determined as the reciprocal of the highest serum dilution that reduced the viral focus counts by 50%.

## 3. Results

### 3.1. SFTSV Infections in Cats in Nagasaki

**Figure 2:**
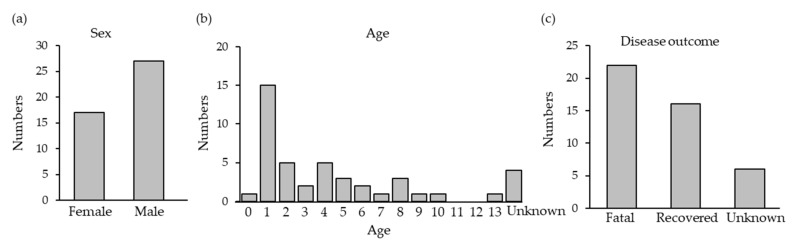
Features of the cats confirmed to have SFTS. (**a**) Sex; (**b**) age; (**c**) disease outcome.

SFTSV RNA was detected in 44 of the 133 cats with suspected SFTS. The SFTSV-positive cats were widely distributed across Nagasaki (Figure 1). The SFTSV-infected cases included 17 females and 27 males with a mean age of 3.5 years, and the fatality rate was 58.0% (22 out of 38 cats except unknown cases) (Figure 2a–c).

In the serum, oral swab, rectal swab, and ocular swab, SFTSV RNAs were detected from 41, 22, 18 and 4 samples, respectively (Table 1). Although the numbers of examined swab samples were limited, most cats that were swab positive were serum positive, and two animals were swab positive but serum negative. These observations raised the concern that the body fluids of SFTSV-infected cats contained SFTSV.

**Figure 3:**
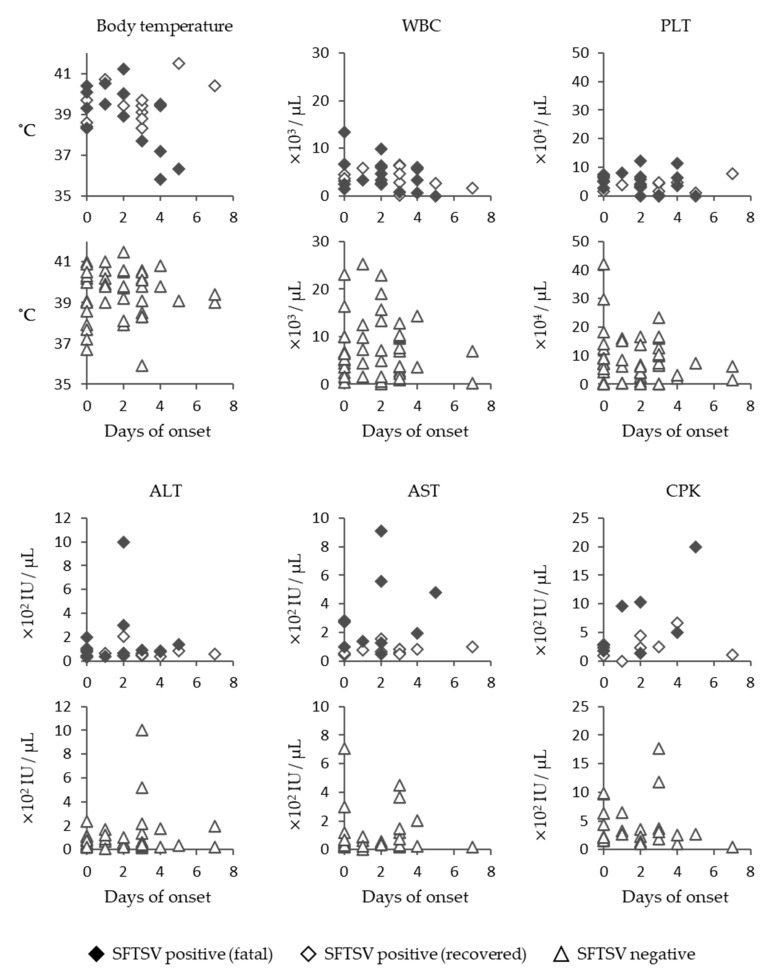
Clinical data of SFTS-infected cats. Body temperature; white blood cell (WBC) count; platelet (PLT) count; and ALT, AST, and CPK levels of SFTSV-positive and -negative cats are indicated.

SFTSV-infected cats showed a high fever in the early days of onset, and in some of the fatal cases, a decrease in the body temperature was seen before death (Figure 3). SFTS cases tended to show lower levels of white blood cells and platelets, and some cats exhibited very low levels, with cell counts below 100 counts/μL (Figure 3). The level of aspartate aminotransferase (AST) in fatal cases tended to be higher than in those of recovered cases (Figure 3). However, substantial increases in alanine aminotransferase (ALT), AST, and creatine phosphokinase (CPK) levels in SFTSV-infected cats were not observed compared with SFTS-negative animals (Figure 3).

**Figure 4:**
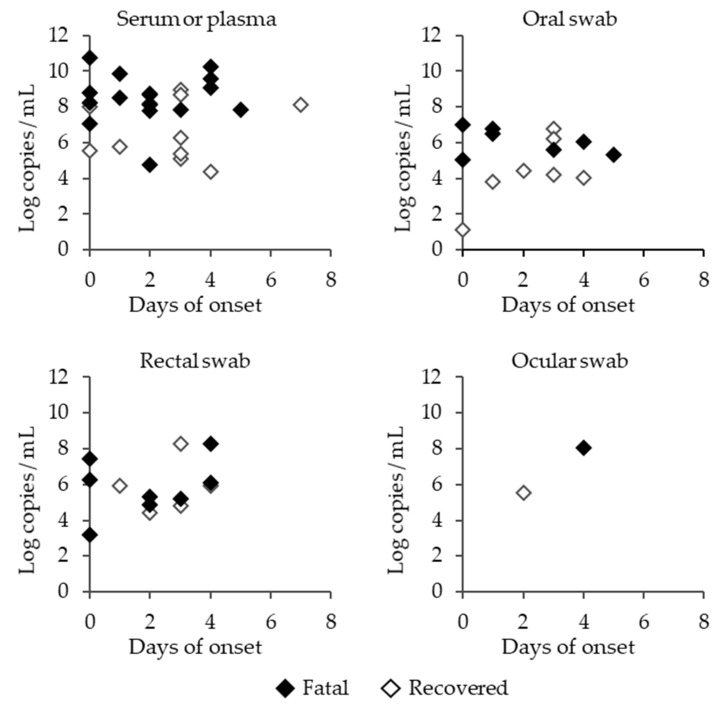
SFTSV RNA copy numbers in the serum or plasma, and oral, rectal or ocular swabs of SFTSV-infected cats. Closed rhombi, open rhombi, and open triangles indicate the fatal cases of SFTSV-infected cats, recovered cases of SFTSV-infected cats, and SFTSV-negative cats, respectively.

High viral RNA levels were detected in the serum during the course of the disease, and they tended to be higher in the fatal cases than in the recovered cases (Figure 4). Remarkably, viral RNAs were detected in the oral, rectal, and ocular swabs, indicating that body fluids, such as saliva, stool, and tears, contain SFTSV. This is an important finding, as it suggests that there is a high risk of infection while providing care to SFTSV-infected animals.

### 3.2. Genetic Background of SFTSV Strains in Nagasaki

**Figure 5:**
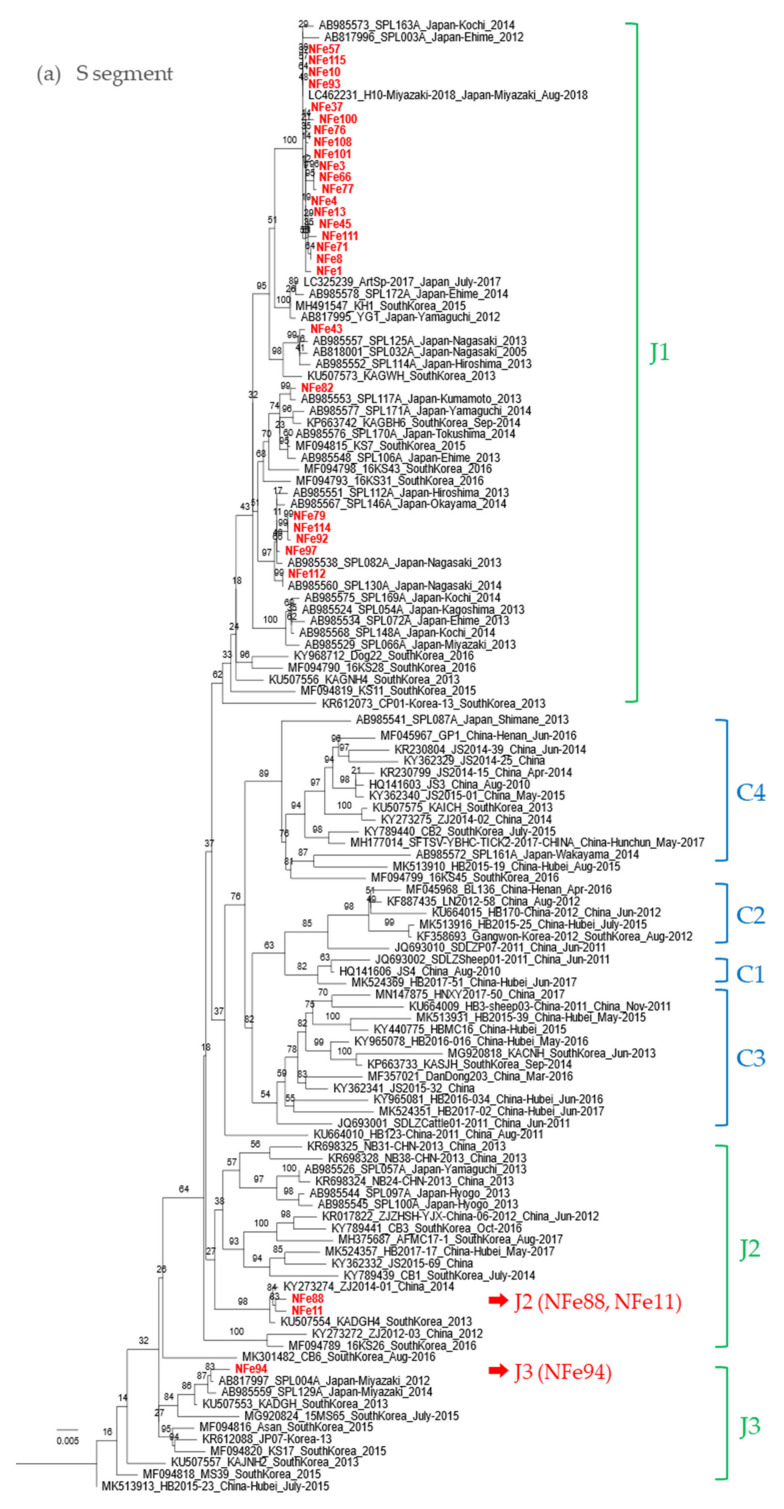

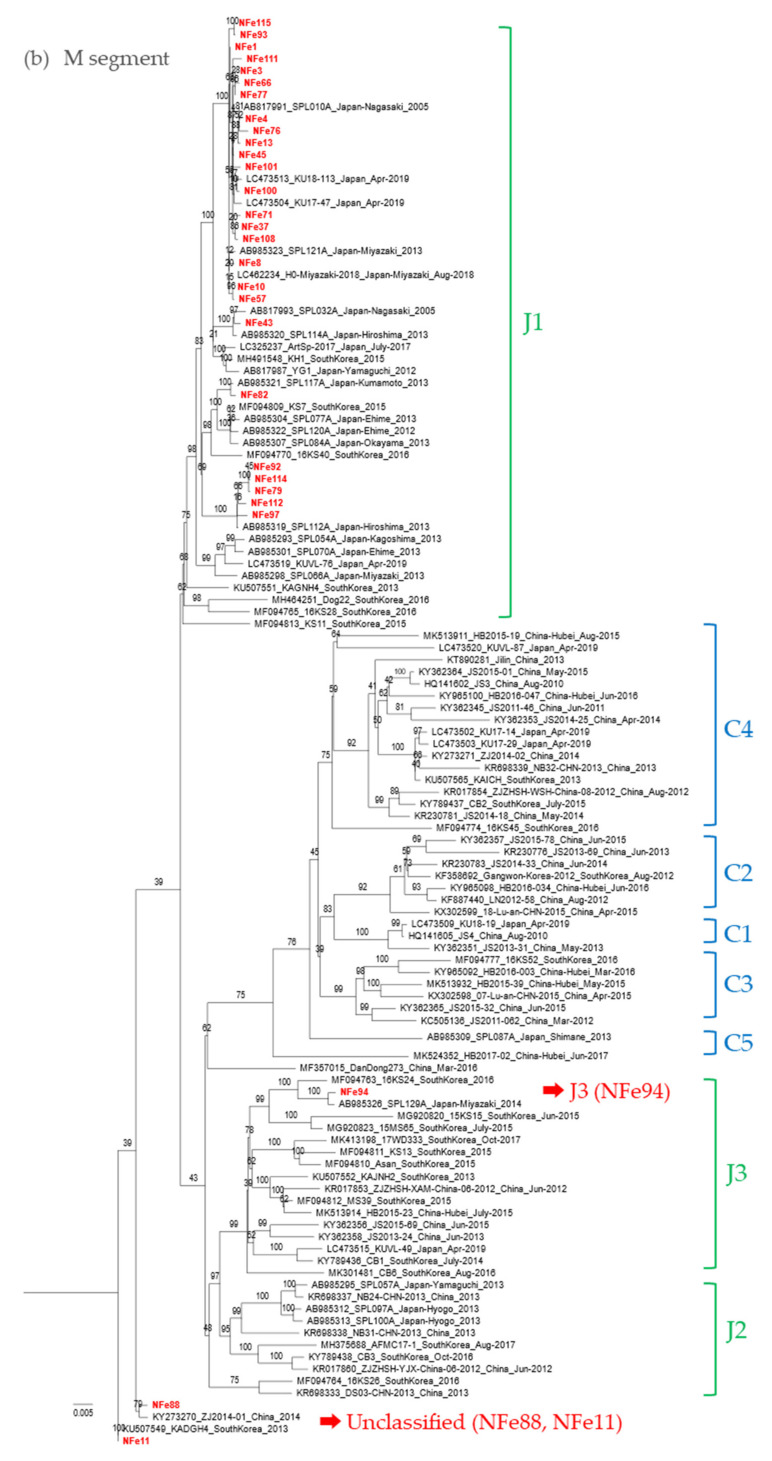

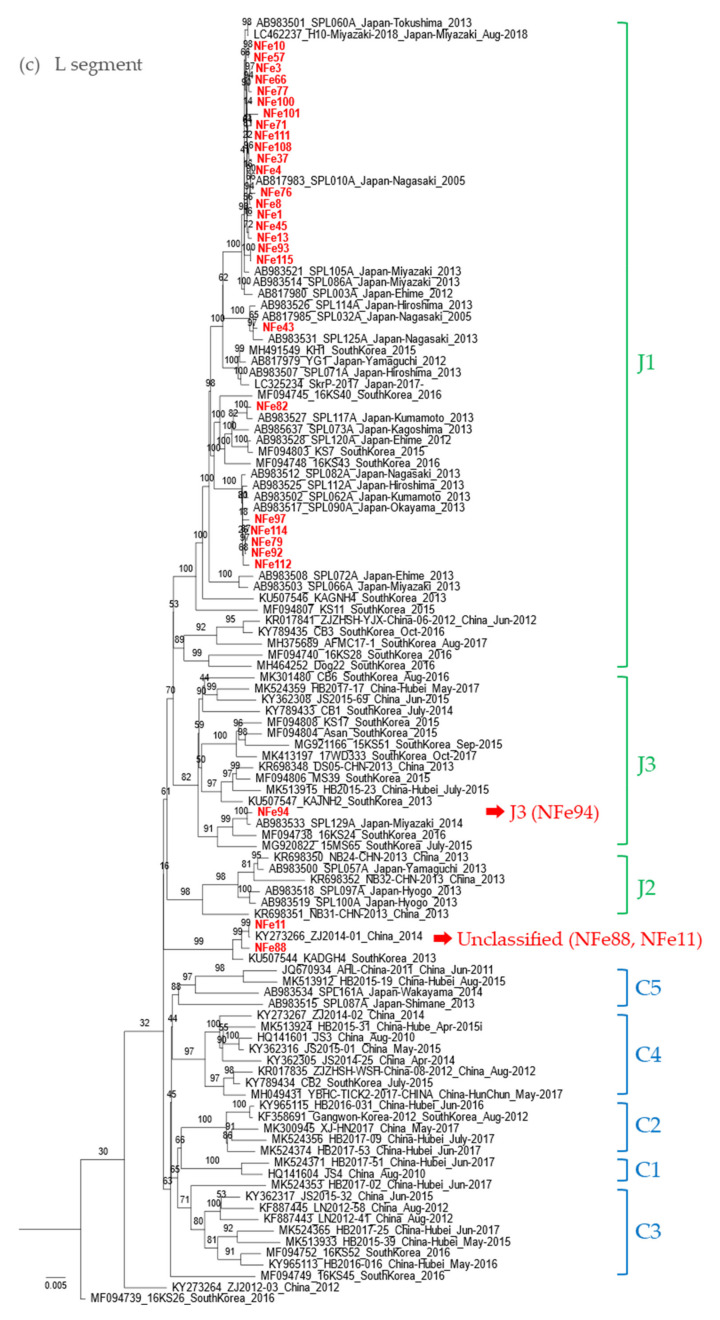
Phylogenetic trees based on the full length of the SFTSV nucleotide sequences for the (**a**) S segment, (**b**) M segment, and (**c**) L segment. The Guertu virus, which belongs to the family Phenuiviridae and genus *Bandavirus*, was used as an outgroup to construct each phylogenetic tree (not indicated in the figures).

To understand the evolutionary relationship between the SFTSV strains in cats, viral sequences of the S, M, and L segments from 30 SFTSV isolates from cats were determined by NGS and were phylogenetically analyzed (Figure 5).

While most strains isolated in this study were classified as genotype J1 in the S, M, and L segments, the NFe94 strain was classified as genotype J3 based on a previous report by Yoshikawa et al. (Figure 5a–c) [28]. Identities of nucleotide sequences between cat-derived strains and reference strains within genotype J1 were more than 96.9%, 96.3%, and 96.4% similar in S, M, and L segments, respectively. Identities of genotype J3 between NFe94 strain and reference strains were more than 97.7%, 96.3%, and 96.8% in S, M, and L segments, respectively.

Interestingly, the NFe88 and Nfe11 strains showed different genotypes in each segment similar to ZJ2014-01 and KADGH4 strains isolated in China and Korea, respectively [29,30]. The S segments were classified as J2 (Figure 5a), the M segments were classified as outside of the clusters of the J and C genotypes (Figure 5b), and the L segments were also classified as an original cluster (Figure 5c). Thus, it appears that the NFe88 and NFe11 strains were reassortant viruses with the J genotype, C genotype, and a possible ancestor genotype of those strains.

### 3.3. Pathogenicity of the SFTSV Strains Isolated from Cats

We next compared the pathogenic potential of the SFTSV strains isolated from cats to examine whether specific strains cause high fatality in cats. The SFTSV strains from fatal cases (strains NFe43, NFe76, NFe82, NFe93, NFe114, and NFe115) and recovered cases (strains NFe4, NFe8, NFe11, NFe77, NFe111, and NFe112) were compared in terms of the virus growth curves in cell cultures and the survival curves of infected A129 mice.

**Figure 6:**
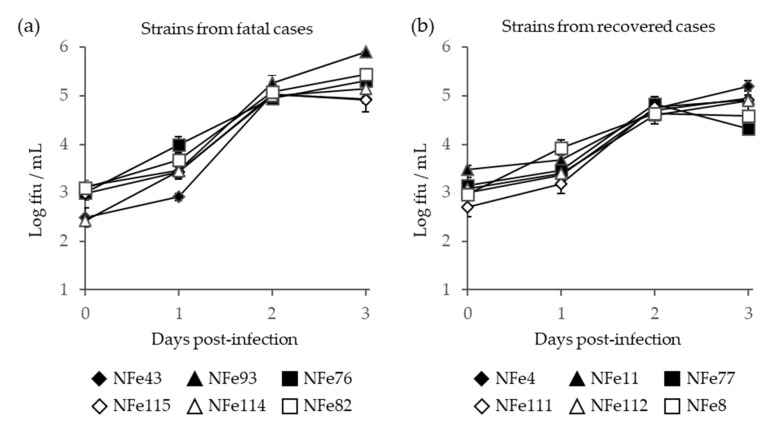
Growth curve of SFTSV in the supernatant of Vero E6 cells infected with SFTSV strains that were isolated from fatal (**a**) and recovered (**b**) cat cases. Error bars indicate the standard deviations.

After infection in Vero E6 cells, the viral titers in the supernatants continuously increased for 3 days; however, there were no significant differences in the growth curves between the SFTSV strains isolated from the fatal and recovered cases (Figure 6).

**Figure 7:**
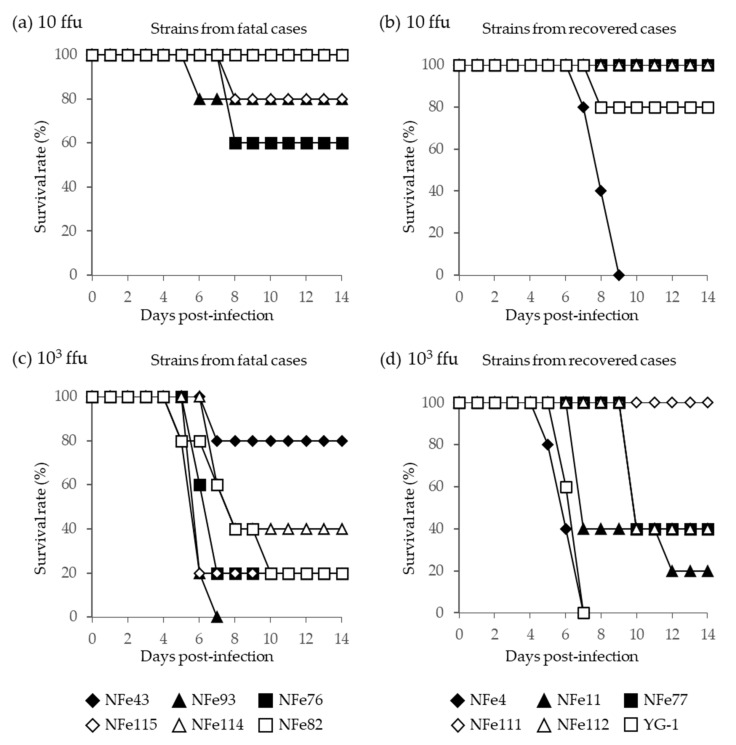
Survival curves of A129 mice infected with (**a**,**b**) the lower dose (10 ffu) and (**c**,**d**) the higher dose (10^3^ ffu) of SFTSV strains isolated from fatal (**a**,**c**) and recovered (**b**,**d**) cat cases. The YG-1 strain isolated from a human case in Japan in 2012 was also compared, and data were presented with strains derived from recovered cats.

In A129 mice, following infection with the lower dose (10 ffu), the fatality rates with the SFTSV strains isolated from the fatal and recovered cases were 0% to 40% and 0% to 100%, respectively (Figure 7a,b). With the higher dose (10^3^ ffu), the fatality rates with the SFTSV strains isolated from the fatal and recovered cases were 20% to 100% and 0% to 100%, respectively (Figure 7c,d). Therefore, the SFTSV strains isolated from the fatal cases did not cause higher mortality in mice when compared to the strains isolated from the recovered cases. In fact, the NFe4 strain derived from a recovered case had a fatality rate of 100% in mice at both 10 and 10^3^ ffu.

From these in vitro and in vivo results, there were no significant differences in the pathogenicity of the SFTSV strains isolated from the fatal and recovered cat cases, suggesting that specific strains of SFTSV are unlikely to cause higher fatality in cats.

### 3.4. Potential Risk of SFTSV Infections in Veterinarians and Nurses

**Figure 8:**
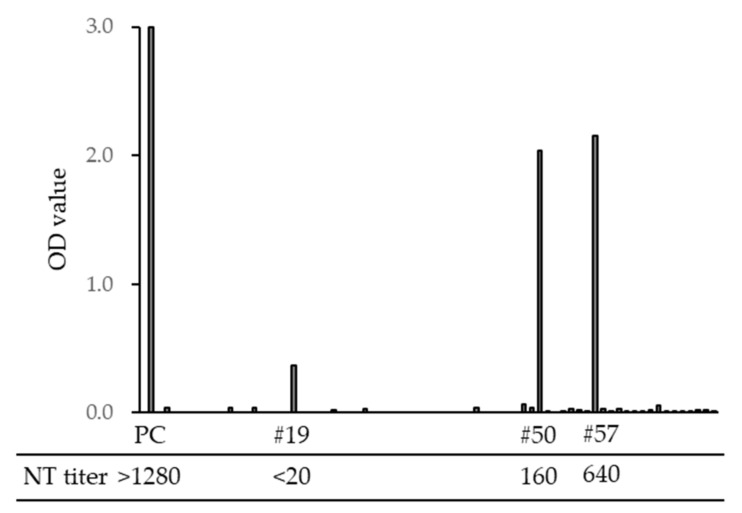
Optical density (OD) value of SFTSV-specific IgG ELISA titers and neutralization (NT) titers of veterinarians and nurses in Nagasaki. PC is a sample from a SFTS-confirmed case. Sample numbers #19, #50, and #57 were IgG ELISA positive, and #50 and #57 were NT positive.

To assess the risk of SFTSV infection when providing animal care, we examined the anti-SFTSV antibody titers of veterinarians and nurses in animal hospitals in Nagasaki prefecture. In total, 3 of 71 veterinary staff members were positive for SFTSV-specific IgG (4.2%); among them, 2 samples exhibited neutralizing titers of 1:160 and 1:640 (Figure 8). In our previous study, we found no anti-SFTSV antibody-positive cases among 326 volunteers, including hunters, soldiers, and famers, who have frequent opportunities to be bitten by ticks [25]; thus, veterinarian staff members appear to have a higher risk of SFTSV infection.

## 4. Discussion

In this study, we reported on SFTS cases among cats. We requested animal hospitals in Nagasaki prefecture to send samples from cats with suspected SFTSV infection, and a total of 133 samples were examined. Among the 133 samples, 44 (33.1%) were confirmed to be infected with SFTSV. Clinical signs of SFTS in cats include fever, leukopenia, and thrombocytopenia, which are similar to the symptoms of human SFTS [1]. Interestingly, most of the cat cases were young, and 1-year-old cats were frequently identified; in contrast, in human cases, an age greater than 50 was a critical risk factor [2,31]. The fatality rate (58.0%) was higher in cats than in humans, indicating that SFTSV is a significant infectious agent that causes severe and fatal infections in cats.

Importantly, our results raised concerns of the high risk of SFTS as a zoonotic infectious disease, because the body fluids of SFTSV-infected animals contained SFTSV. Indeed, there have been some human cases that were suspected to have been infected by SFTSV through contact with cats and dogs with SFTS [24]. Our results and previous studies indicate that veterinarian staff members may become infected with SFTSV after exposure to SFTSV-infected animals [22,23]. Therefore, it is strongly recommended for veterinarians, nurses, and pet owners to take proper prevention measures when caring for animals with SFTS or suspected SFTSV infection.

SFTSV infections in animals have been studied ever since the first human case of SFTS was reported, although it was unknown whether animals showed clinical signs or died due to the virus. The first animal SFTS cases in cheetahs were reported in 2017 in Japan [19], and some SFTS cases were also identified in cats and dogs [14,20,21]. These reports indicated that animals are at a risk of infectious disease due to SFTSV.

From 2018 to 2020 in Nagasaki, there were 47 confirmed SFTS cases in cats, including this study and data from personal communication, and 18 confirmed SFTS cases in humans, indicating that cat cases are more frequently identified than human cases. SFTSV can be isolated from infected humans and animals, although virus isolation from ticks and asymptomatic animals is limited [26]. In addition, personal information, such as an individual’s place of residence, living or deceased status, and age, is sometimes not available due to privacy concerns in Japan. Therefore, cats can be considered useful as sentinel animals for investigating the features of the virus, such as the phylogenetic and geographical distribution in endemic areas, and the pathogenicity based on the SFTSV strains isolated from cats.

In this study, 30 strains of SFTSV were isolated from cat samples, including fatal and recovered cases. SFTSV-infected cats were distributed across a wide area of Nagasaki. Most SFTSV strains were classified as genotype J1, suggesting that genetically close strains of J1 are mainly distributed in Nagasaki. Genotypes of SFTSV strains were first presented by Yoshikawa et al. [28], and other groups also reported and suggested additional genotypes [29,30,32,33]. In addition, it has been suggested that reassortants of SFTSV are key factors driving increasing genetic diversity, raising concerns of antigenicity and pathogenicity alterations [29,30].

Interestingly, the NFe88 and Nfe11 strains were reassortant viruses, and they were clustered together with ZJ2014-01 and KADGH4 strains isolated in China and South Korea, respectively [29,30]. Although these strains were classified as original genotype clades in previous reports [29,30], the genotypes of these strains were J1 in the S segment and unclassified in the M and L segments. These two strains were isolated from cats that were located close to each other in the Sasebo area. Although it is currently unknown how and where the reassortment occurred for the original NFe88 and NFe11 strains, further phylogenetic and geographic surveys may provide clues for elucidating how SFTSV spreads in endemic areas in Japan, China, and Korea.

To examine whether the high severity of the disease in cats is due to the pathogenicity of specific virus strains, we compared the growth and pathogenic properties of SFTSV isolates from fatal and recovered cat cases. However, there were no significant differences in the virus growth curves in susceptible Vero E6 cells nor in the fatality rates of infected A129 mice between the strains isolated from fatal and recovered cat cases. In fact, the NFe4 strain that was isolated from a recovered case showed high pathogenicity. Thus, it is unlikely that the high pathogenicity is due to viral factors of specific strains of SFTSV. Further elucidation focusing on host factors specific to cats will be required and may provide useful information about the high pathogenicity of SFTSV specific to cats.

## 5. Conclusions

Our results show that SFTSV is an important infectious agent that causes severe and fatal infections in cats, and that more cases in cats have been identified compared to human cases in Nagasaki. Thus, we suggest that cats are a useful sentinel animal for the study of SFTS. Importantly, it was shown that the body fluids of SFTSV-infected animals contain SFTSV, suggesting that there is an increased risk of infection while providing care to SFTSV-infected animals. Veterinarian staff showed higher seroprevalence of SFTSV, thus it is strongly recommended that proper prevention measures are taken when providing care to SFTSV-infected and SFTS-suspected animals.

## Figures and Tables

**Table 1:** Numbers of SFTSV RNA detection in the samples of SFTSV-infected cats.

	Serum	Oral Swab	Rectal Swab	Ocular Swab
SFTSV RNA detected	41	22	18	4
SFTSV RNA not detected	2 ^2^	3	1	1
Not examined ^1^	1 ^3^	19	25	39

^1^ Samples were not provided. ^2^ These animals were SFTSV RNA positive in the oral and rectal swabs. ^3^ This animal was SFTSV RNA positive in the oral, rectal, and ocular swabs.
